# The Benefit of Air Conduction Pure-Tone Audiometry as a Screening Method for Hearing Loss over the VAS Score

**DOI:** 10.3390/diagnostics14010079

**Published:** 2023-12-28

**Authors:** Aris I. Giotakis, Lambros Mariolis, Ioannis Koulentis, Christos Mpoutris, Evangelos I. Giotakis, Aikaterini Apostolopoulou, Efstathios Papaefstathiou

**Affiliations:** 1 First Department of Otorhinolaryngology Head and Neck Surgery, Metropolitan General, 15562 Athens, Greece; 2 First Department of Otorhinolaryngology, Hippocrateion General Hospital, 11527 Athens, Greece; 3 Laboratory of Hygiene, Social & Preventive Medicine and Medical Statistics, School of Medicine, Aristotle University of Thessaloniki, 54124 Thessaloniki, Greece; katapost@yahoo.gr; 4 Second Department of Urology, Aristotle University of Thessaloniki, General Hospital ‘Papageorgiou’, 56403 Thessaloniki, Greece; epapast@hotmail.com

**Keywords:** hearing loss, mass screening, audiometry, young adult, methods

## Abstract

Hearing loss is commonly encountered by general practitioners. We aimed to evaluate the screening benefit of air conduction pure-tone audiometry over visual analogue scale (VAS) scores for hearing loss. Moreover, we intended to perform the first cross-sectional study in Greece to assess hearing loss with pure-tone audiometry in young adults of the general population. We evaluated Greeks between 15 and 40 years old in a high school in Karditsa, Greece, and a primary health care unit in a nearby community. Subjects filled out a VAS score sheet and underwent pure-tone audiometry in a room without sound isolation, with air conduction only. We named the latter procedure modified pure-tone audiometry (mPTA). Subjects with pathologic results were examined via otoscopy and standardized pure-tone audiometry (sPTA). Of the 286 subjects evaluated, the VAS score revealed 5 subjects (1.7%) with hearing loss. mPTA (100 s duration) doubled this percentage (in total 3.8%; Pearson Chi-Square test; *p* < 0.001). Based on sPTA, the sensitivity and positive predictive value of the VAS score were 40% and 13%, respectively. For mPTA, they were 100% and 37%, respectively. mPTA filtered out pathologic cases in a proper, rapid, cheap and simple way and may be considered a proper screening method for hearing loss in primary health care.

## 1. Introduction

Approximately 12% of adults aged <40 years report difficulties following a conversation if there is background noise, according to the Center for Disease Control and Prevention of the United States of America. This percentage increases to 42% for patients aged >70 years. Hearing loss is defined by a hearing threshold (500, 1000, 2000 and 4000 Hertz) of 25 decibels (dB) or more [1], whereas disabling hearing loss usually refers to hearing loss greater than 41 dB in the better hearing ear. The estimated number of people with disabling hearing loss in Greece is approximately one million (prevalence of 10%). However, this is only an estimation from Greek authorities based on European statistics [2].

Hearing loss is associated with social isolation and loneliness [3,4,5,6] or even Alzheimer’s disease [7]. The relationship of hearing loss with social isolation and loneliness is supported by a large amount of data from national studies [8,9,10]. Specifically, Huang and coauthors investigated 933 subjects. The authors reported that worse hearing was associated with 19% greater prevalence of moderate or greater loneliness (prevalence ratio: 1.19; 95% confidence interval: 1.06, 1.33). Also, a worse hearing-related quality of life was associated with a 29% greater prevalence of moderate or greater loneliness (prevalence ratio: 1.29; 95% confidence interval: 1.19, 1.39) and worse social network characteristics (e.g., more constricted social network size (incidence rate ratio: 0.96; 95% CI: 0.91, 1.00)) [8]. Therefore, early identification of this condition is important [8,11].

Screening methods include questionnaires as well as subjective and objective tools. Questionnaires for diagnosis of hearing loss include, among others, the visual analogue scale (VAS) score test [12]. Bokari and coauthors investigated 160 subjects and reported that the VAS can be best used in young adults. The authors concluded that the VAS may be applicable in rural settings as a screening procedure when audiometry is not available. It can enhance clinical hearing assessments, especially in mild-to-moderate conductive hearing loss. It can also help the clinician to gauge the degree of handicap the patient feels due to their hearing loss [12].

Among subjective tools, pure-tone audiometry (PTA) [13] is the main test for evaluations of hearing loss. Pure-tone (isolated frequency) audiometry evaluation over the range of frequencies important for everyday listening can determine the degree, configuration and type of hearing loss in a manner detailed enough to assist a health care team in determining the etiology and prognosis for hearing loss, as well as the optimal treatment strategy [14]. Although VAS tests are simple and time-efficient screening methods for evaluations of hearing loss in primary health care, their accuracy might be limited when used alone [15]. Louw and coauthors investigated 1084 subjects. Of them, 40% self-reported hearing loss. However, only 12.5% self-reported hearing loss and failed audiometry tests. The authors concluded that combination with PTA can significantly improve the efficiency of the VAS in evaluations of hearing loss [16].

Evaluation of hearing loss is commonly performed by general practitioners [17]. Still, several challenges may emerge. PTA might require specific facilities [18], such as a soundproof, isolated room. Moreover, the examination might last 15 min per patient.

Sufficient, yet rapid, screening for hearing loss in primary health care is important. With this study, we aimed to evaluate the exact advantage of performing only the air-conduction part of PTA in a room without sound isolation over VAS tests for hearing loss in Greek young adults. Our final purpose was to suggest a proper, rapid, cheap and simple screening method to be used in primary health care for evaluations of hearing loss.

## 2. Materials and Methods

### 2.1. Study Population and Design

In a cross-sectional study, Greeks aged between 15 and 40 years old, examined during screening in a high school in Mouzaki, Karditsa, Greece, and a primary health care unit in a nearby community, were eligible. Screening was performed by five members of the First Department of Otorhinolaryngology Head and Neck Surgery, Metropolitan General, Athens, Greece, who were invited by members of the local authorities. The invitation was non-profit. Examinations lasted a single day per unit. The study protocol was approved by the Ethical Committee of Metropolitan General (24-01-2023/500) and the Greek Ministry of Education and Religion.

Data acquisition was performed with a questionnaire including the visual analogue scale score for hearing and pure-tone audiometry for evaluation. The duration of the examination was measured in a few subjects with a stopwatch on a smartphone owned by the examiners. Only the duration of the examination of subjects with normal hearing was documented.

### 2.2. Examination Room

On the first day, we examined pupils aged 15 to 18 years old, in their high school facilities. Examinations were performed in a separate, non-isolated, but quiet classroom at the end of a corridor on the second floor. Pupils were gathered at the beginning of the corridor, approximately 50 m away from the examination room. In the examination room, two examiners sat on two chairs next to each other behind a desk. The audiometer was placed on the desk in front of the examiners. The VAS documentation paper was on the right of the audiometer. The audiometry documentation paper was on the right of the VAS document. The examination chair was placed in front of the desk. Each study subject was sitting on the chair with their back facing the examiners. The door was closed during the examination. One examiner asked the subjects about their hearing and performed the hearing test, while the other examiner noted the results with a pen and was responsible for the measurement of the examination’s duration. The duration was measured for every third subject and it was documented only in subjects with normal hearing. School teachers were responsible for the entry and exit of pupils in and out of the examination room.

On the second day, we examined adults aged 18 to 40 years old in the primary health care unit of their community located 10 km away. In the primary health care unit, a similar setting was used. The examination room was a simple examination room located next to the lobby. Here, one member of the primary health care unit’s staff was responsible for the entry and exit of subjects into and out of the examination room.

Results were transferred to an Excel sheet the day after the examinations.

### 2.3. Visual Analogue Scale Score

Subjects were first asked to evaluate their hearing using the VAS score [12]. The VAS score comprises a 5-point rating scale, as follows: normal hearing or mild, moderate, severe and profound hearing loss correspond to 1, 2, 3, 4 and 5, respectively [12]. Answers were noted for each ear separately. The documentation paper was a non-validated questionnaire. Specifically, the documentation paper was an A4 size piece of paper with a table consisting of 3 columns and 40 rows. Multiple documentation papers were available during the examination. The first, second and third column in the first row included a serial number, starting from one, and the word “right” and the word “left”, respectively. Right and left refer to the right and left ear, respectively. When the subject sat on the examination chair, we asked how the subject would evaluate their hearing. If the subject said “unproblematic” or something similar, we would note “1” in both the second and third columns. If the subject said “I’m having some hearing problems”, we would then ask if both ears were affected and if not both, then which ear. We would then further ask if the subject could describe their hearing loss as mild, moderate, severe, or profound. We noted the answer accordingly.

### 2.4. Modified Pure-Tone Audiometry

For audiometry, a fully portable automatic AS216 audiometer from Interacoustics (Middelfart, DK 5500, Denmark) with a standard TDH39 headset and an APS2 patient response button was used. This was a 30 × 23 × 9 cm (width, depth, height) device with a weight of 1.3 kg, an external power supply and a numerical LCD display. Several buttons were present on the main surface. The main buttons were, from left to right, the talk forward button, the decibel adjustor, the tone switch, the tone character (e.g., pulsed or continuous) button and the frequency adjustor. Eleven frequencies from 125 Hertz (Hz) to 8 kHz with a −10 dB to 120 dB output were available. The right ear was first examined at six frequencies (500 Hz and 1, 2, 3, 4 and 6 kHz) with a pulsed tone, followed by the left ear in a similar manner. We used only the air conduction and not the bone- onduction part. This procedure was defined as “modified pure tone audiometry (mPTA)”. The results were noted for each ear separately as follows: the numbers 1, 2, 3, 4 and 5 correspond to 0–25 dB, 26–40 dB, 41–60 dB, 61–80 dB and >80 dB levels. Here, we noted the worst hearing threshold among all frequencies; e.g., if a subject’s hearing threshold was 0–25 dB at 5/6 frequencies and 41–60 dB at a single frequency, then the number 3 was noted. The above-mentioned numbers 1–5 were also noted per examined frequency, but only in the first three hours of each examination day.

### 2.5. Definition of Hearing Problems and Pathologic mPTA

Concerning the absence of an otoscopic examination, external sound isolation and standardized pure-tone audiometry (sPTA), subjects with a hearing threshold between 25 and 40 dB in at least one ear and one examined frequency were informed that they had a low risk of a current “hearing problem” and were encouraged to visit an Otorhinolaryngologist in the future for an otoscopic examination and sPTA. On the contrary, subjects with a high risk of a disabling hearing loss (hearing threshold of 41–60 dB and higher) in at least one ear and at one examined frequency were thought to suffer indeed from a “hearing problem” and were labeled with pathologic mPTA.

### 2.6. Standardized Pure-Tone Audiometry

Subjects who reported a mild, moderate, severe, or profound hearing loss in the VAS score and subjects with pathologic mPTA underwent an otoscopic examination and sPTA. Results were cross-checked.

### 2.7. Outcome Parameters

We intended to assess the percentages of VAS scores, hearing loss based on mPTA and hearing loss per frequency. Moreover, we evaluated the homogeneity of the mPTA results. The mPTA results were considered homogeneous if the same hearing threshold was observed in all frequencies. Also, we noted how often a frequency was different from the other frequencies. Lastly, we calculated the accuracy of the VAS and mPTA based on formulas of Taylor and Emanuel [19], considering sPTA as the reference method.

### 2.8. Statistics

Data were analyzed with the SPSS 26.0 statistics package (SPSS Inc., Chicago, IL, USA). Count data were tabulated; for metric data, means and standard deviations were calculated. The normality of the distribution of variables was tested with the Kolmogorov–Smirnov test or Shapiro–Wilk test, depending on the study sample size. For comparison of categorical variables, the Pearson Chi-Square test was used. Results were considered significant if *p*-values were lower than 0.05.

## 3. Results

### 3.1. Subject Population

In total, 286 subjects participated in the study. One hundred and fifty were women. The median age was 26 years (range: 15–40 years; lower to upper quartile 21 to 32 years). Data per frequency were available for 106 subjects. The duration of the examination per subject with normal hearing was approximately 100 s.

### 3.2. VAS Scores for Diagnosis of Hearing Loss

**Figure 1:**
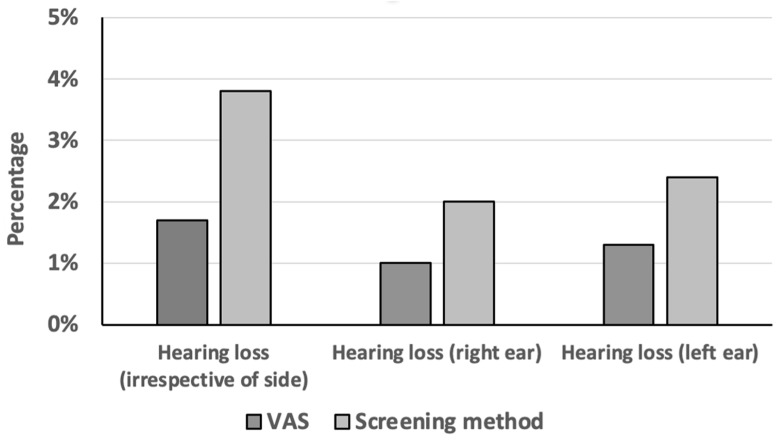
Comparison of results obtained using VAS scores and the screening method in 286 subjects. *y*-axis: percentage; *x*-axis: hearing loss; e.g., five (1.7%) subjects reported hearing loss according to the VAS score, while modified pure-tone audiometry revealed 11 subjects (in total 3.8%) with hearing loss.

Of the 286 subjects, 5 subjects (1.7%) reported hearing loss (Figure 1). Of them, three (1.0%) and two (0.7%) subjects reported mild and moderate hearing loss, respectively. Only two out of five subjects reported a hearing loss in both ears, which was mild.

### 3.3. Subjects with Pathologic Modified Pure-Tone Audiometry

The mPTA revealed 11 subjects (in total 3.8%) with a hearing problem, thus doubling the percentage of subjects with a hearing problem compared to the VAS score (Figure 1; Pearson Chi-Square test; *p* < 0.001). Interestingly, mPTA in two subjects with a pathologic VAS score was normal.

**Figure 2:**
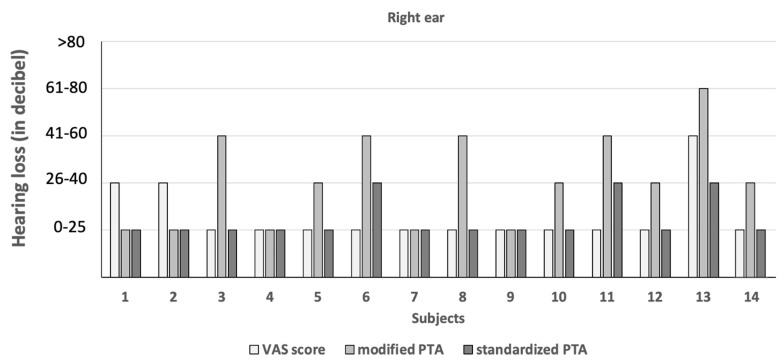
Comparison of hearing loss between hearing evaluation methods in subjects using the pathologic visual analogue scale (VAS) score or pathologic modified pure-tone audiometry (PTA) in the right ear. *x*-axis: subjects. *y*-Axis: hearing loss in decibels. Hearing evaluation methods: white: VAS score; light grey: modified pure-tone audiometry; dark grey: standardized pure-tone audiometry. For example, in the VAS score, subject 6 reported no hearing loss, while modified pure-tone audiometry revealed a moderate hearing loss (41–60 dB). However, only a mild (26–40 dB) hearing loss was found by standardized pure-tone audiometry.

**Figure 3:**
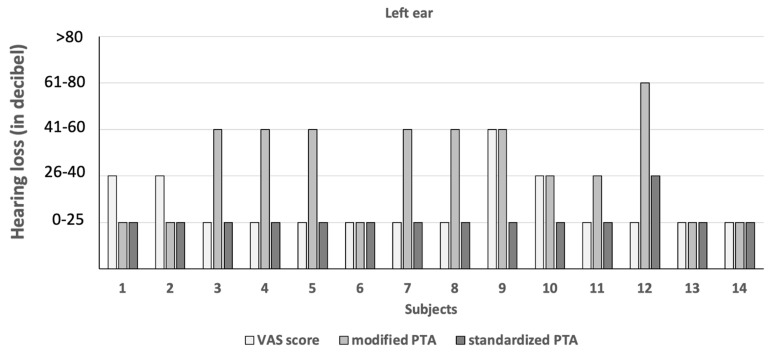
Comparison of hearing loss between hearing evaluation methods in subjects using the pathologic visual analogue scale (VAS) score or pathologic modified pure-tone audiometry (PTA) in the left ear. *x*-axis: subjects. *y*-axis: hearing loss in decibels. Hearing evaluation methods: white: VAS score; light grey: modified pure-tone audiometry; dark grey: standardized pure-tone audiometry. For example, in the VAS score, subject 12 reported no hearing loss. On the contrary, modified pure-tone audiometry revealed a severe (61–80 dB) hearing loss. However, only a mild (26–40 dB) hearing loss was found by standardized pure-tone audiometry.

The hearing thresholds of the 11 subjects in pathologic mPTA were 41–60 dB (right ear: 1.7%; left ear: 2.1%) and 61–80 dB (right ear: 0.3%; left ear: 0.3%; Figure 2 and Figure 3). The hearing threshold of 26–40 dB was by definition not pathologic, and it was noted in the right ear of 46 subjects (16.1%) and the left ear of 46 subjects (16.1%).

### 3.4. Screening Characteristics of Hearing Evaluation Methods

The VAS score, mPTA and sPTA revealed five, eleven and four (1.4%) subjects with a hearing problem, respectively (Figure 2 and Figure 3). The VAS scored low in sensitivity (<50%) and high regarding the false negative rate (>50%; Table 1). The over-referral rate was higher for the VAS (75–100%) than for mPTA (40–86%; Table 1). The positive predictive value was lower for the VAS (<25%) than for mPTA (14–60%; Table 1). In contrast to the VAS, the mPTA test scored better in screening characteristics (Table 1).

In both ears, the most frequently observed normal frequency was 1000 Hz (right ear: 100%; left ear: 98.1%), followed by 3000 Hz (right ear: 99.1%; left ear: 98.1%) and 2000 Hz (right ear: 98.1%; left ear: 97.2%). The most frequently observed pathological frequency was 6000 Hz (right ear: 7.5%; left ear: 8.5%), followed by 4000 Hz (right ear: 3.8%; left ear: 5.7%). We observed no significant differences between sides (all Pearson Chi-Square tests; *p* > 0.2; Table 2). We noted a higher hearing loss at 4000 Hz compared to that at 6000 Hz in only one subject (0.9%).

### 3.5. Homogeneity of Modified Pure-Tone Audiometry Results

**Figure 4:**
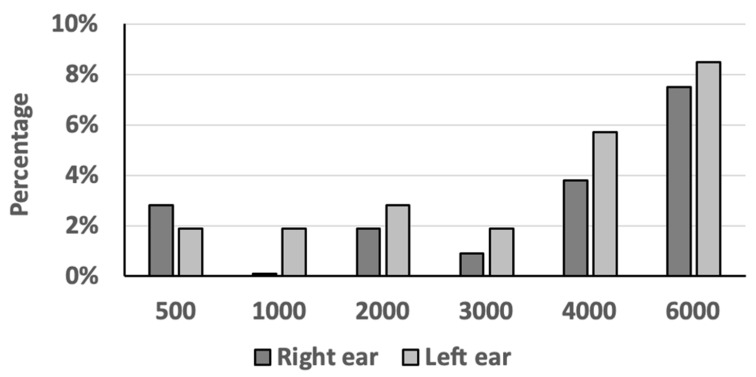
Comparison of heterogeneity of modified pure-tone audiometry results between the right and left ear per frequency in 106 subjects. *y*-axis: percentage; *x*-axis: frequency (Hz). For example, the frequencies 500 Hz and 6000 Hz were different from all the other frequencies in 2.8% and 7.5% of the subjects in the right ear, respectively, and 1.9% and 8.5% of the subjects in the left ear, respectively.

We noted 13 (12.3%) heterogeneous mPTA results in the right ear and 16 (15.1%) in the left ear. The hearing threshold at 1000 Hz was the frequency that most often did not differ from the other frequencies (right ear: 0%; left ear: 1.9%), followed by 3000 Hz (right ear: 0.9%; left ear: 1.9%; Figure 4). As expected, the hearing threshold at 6000 Hz was the frequency that most often differed from the other frequencies (right ear: 7.5%; left ear: 8.5%), followed by 4000 Hz (right ear: 3.8%; left ear: 5.7%; Figure 4).

## 4. Discussion

There is a paucity of data on the benefit of PTA over questionnaires in primary health care. During evaluations of hearing loss, accurate and fast examination of a large number of subjects in a simple way is essential. Our final purpose was to suggest a proper, rapid, cheap and simple screening method to be used in primary health care. Initially, we intended to assess this screening test in young adults <40 years old, where the probability of a hearing loss is sufficiently lower compared to adults >40 years old.

In this cross-sectional study, we used a pure-tone audiometry without bone conduction test in a room without sound isolation (mPTA). Our results supported the rapid character of this screening test, since the duration of the examination was approximately 100 s per subject. Also, its simplicity was confirmed by the absence of special facilities.

mPTA doubled the percentage of young adults with hearing loss to 3.8% compared to the 1.7% for the VAS score. However, cross-checking with sPTA revealed a low positive predictive value for both tests (Table 1). This implied that only 1/8 of patients with pathologic VAS scores (approximately 13%) and 3/8 of patients with pathologic mPTA scores (approximately 37%) indeed suffered from hearing loss. Despite the low positive predictive value, the sensitivity was excellent for mPTA in contrast to the VAS score. However, this was expected. Subjects with a pathologic sPTA score also had a pathologic mPTA score, since only subjects with a pathologic mPTA score underwent sPTA. Despite the low positive predictive value and the expected high sensitivity, mPTA succeeded in filtering out the subjects that needed further examination in a proper, cheap, rapid and simple way. This emphasized its utility in primary health care screening. Future studies could perform sPTA in all subjects in order to be able to assess more screening characteristics, such as specificity, false positive rate, under-referral rate and negative predictive value.

Furthermore, sPTA revealed hearing loss in 1.4% of the subjects. These data were representative for the young population in Greece. The number of infants, children and adolescents with hearing loss in Greece is estimated at 100,000, which accounts for 1% of the general population. In Greece, there is a significant paucity of studies that assess hearing loss via PTA in young adults of the general population. On the contrary, several studies have assessed hearing loss in several subgroups of patients [20,21,22]. This study may well be considered as the first cross-sectional study in Greece to assess hearing via PTA in young adults without known hearing problems.

Data on the comparison of VAS scores and PTA are sparce. Bokari and coauthors searched for cases where the results of the VAS and PTA matched and reported a rather poor correlation (or agreement) between both methods [12]. Moreover, several studies have described air conduction PTA [23,24] in a room without sound isolation [18,25,26]. Specifically, Maclennan-Smith and coauthors concluded that valid diagnostic pure-tone audiometry can be performed in a natural environment with recently developed technology, offering the possibility of access to diagnostic audiometry in communities where soundproof booths are unavailable [26]. However, studies suggesting the use of air conduction PTA in a room without sound isolation, i.e., mPTA, as a screening method in primary health care were hard to find.

The mPTA setup was simple for the investigators. Its limitations compared to the established standardized version included the absence of a soundproof, isolated room [27] and the lack of a previous clinical examination by an ENT doctor [24]. A proper screening test should identify subjects with potential hearing loss. Therefore, the screening test aimed to have a high specificity and high negative predictive value. The threshold of 40 dB in the air conduction part without sound isolation would reveal most cases with hearing loss. The bone conduction part, which examines the inner ear and central pathway, could be avoided, since it cannot be worse than the air-conduction part [28]. This strategy, i.e., air conduction only with a threshold of 40 dB without sound isolation, would identify all subjects with hearing loss. On the contrary, it would also identify subjects with easily reversible causes of hearing loss, such as cerumen impaction. This condition is easily treated in an outpatient clinic within minutes [29]. Thus, cerumen impaction is not considered as clinically important hearing loss. Such cases could partly explain the relatively low positive predictive value of mPTA. Other causes may include stress, concentration problems and mental fatigue.

As expected, the most often solely pathologic frequency was 6000 Hz. Interestingly, we noted a tendency of the higher frequencies to be more often solely pathologic than the lower frequencies (Figure 4). This could imply that examination of higher frequencies, i.e., 4000 and 6000 Hz, may suffice in times of need, since the probability of the lower and middle frequencies, i.e., 500 to 3000 Hz, of being solely pathologic is lower than 3%. The probability of frequencies of 4000 and 6000 Hz being solely pathologic increased to 4% and 8%, respectively. These findings may be important in primary health care units throughout the world, where the availability of personnel, equipment and time is limited [30,31]. This also may emphasize the importance of the 8000 Hz frequency, which was not examined in this study.

The study results were representative of data observed in the young Greek population. However, this study was performed exclusively in young adults. Future studies could focus on examining more frequencies, i.e., 125, 250 and 8000 Hz, as well as on examining older adults.

This study had several limitations. First, we did not perform sPTA in subjects with a hearing threshold between 25 and 40 dB in mPTA in at least one ear and at one examined frequency. This constitutes a mild hearing loss and should be further examined properly. However, the insufficient personnel resources, the limited time and the restricted examination space did not allow us to perform sPTA in at least 50 subjects. Nevertheless, subjects were encouraged to visit local otorhinolaryngologists for further examination. Second, we did not assess frequencies of 125, 250 and 8000 Hz. The study design included assessment of frequencies from 500 to 6000 Hz due to the study’s exploratory character and limited time. Third, we could have documented other screening characteristics, such as specificity, false positive rate, under-referral rate and negative predictive value, if all subjects underwent sPTA. This would lead to a more complete comparison between VAS scores, mPTA and sPTA. Fourth, the study results are directly applicable to adolescents and adults younger than 40 years. Application to adults older than 40 years may require further study. Fifth, while the study design allowed for replication of the conditions found in a primary health care setting, examination of two different populations, i.e., pupils aged from 15 to 18 years and adults aged from 18 to 40 years in two different places, might lead to systematic bias. Sixth, hearing ability is generally better in women than in men [32], as well as in young people compared to older people. This study did not reveal any age or gender differences. The study population, i.e., young people, might be a reason for this. Lastly, we did not measure the noise level in the examination room [33], which would have been desirable.

## 5. Conclusions

This study should be considered as the first cross-sectional study in Greece to assess hearing using PTA in young adults without known hearing problems. Furthermore, the findings suggested that screening of higher frequencies exclusively, e.g., 6000 Hz, may suffice for recognition of significant hearing loss due to the higher probability of 6000 Hz being solely pathologic compared to the lower frequencies. This would be important for primary health care centers with limited resources. This study managed to describe a proper, rapid, cheap and simple screening method that can be used in primary health care for evaluations of hearing loss. This screening method has a significant benefit over VAS scores and can filter out pathologic cases without the need for trained staff. Due to its simplicity, it can also be easily applied in educational and military institutions. Moreover, mPTA may be used in geriatric institutions to identify cases of significant age-related hearing loss.

## Figures and Tables

**Table 1:** Characteristics of the screening tests for evaluation of hearing loss based on standardized pure-tone audiometry as a reference method.

Accuracy Measure	Right Ear		Left Ear		Probability That … ^1^
	VAS	mPTA	VAS	mPTA	
Sensitivity	33%	100%	50%	100%	Someone with the disorder has a positive screening result
False negative rate	67%	0%	50%	0%	Someone with the disorder has a negative screening result
Positive predictive value	25%	60%	0%	14%	Someone who has a positive screening result has the disorder
Over-referral rate	75%	40%	100%	86%	Someone who has a positive screening result does not have the disorder

^1^ Taylor and Emanuel [19]. VAS: visual analogue scale. mPTA: modified pure-tone audiometry. Hearing loss per frequency.

**Table 2:** Hearing thresholds of modified pure-tone audiometry per ear and frequency in 106 subjects.

Ear	Frequency (Hz)	Decibels					*p*-Value ^1^
		0–25 dB	26–40 dB	41–60 dB	61–80 dB	>80 dB	
Right	500	97.2%	2.8%	0%	0%	0%	
Left		98.1%	1.9%	0%	0%	0%	>0.2 ^2^
Right	1000	100%	0%	0%	0%	0%	
Left		98.1%	1.9%	0%	0%	0%	>0.2
Right	2000	98.1%	1.9%	0%	0%	0%	
Left		97.2%	2.8%	0%	0%	0%	>0.2
Right	3000	99.1%	0.9%	0%	0%	0%	
Left		98.1%	1.9%	0%	0%	0%	>0.2
Right	4000	96.2%	3.8%	0%	0%	0%	
Left		94.3%	4.8%	0.9%	0%	0%	>0.2
Right	6000	92.5%	5.7%	1.9%	0%	0%	
Left		91.5%	5.7%	2.8%	0%	0%	>0.2

^1^ Differences between right and left ears were assessed by a Pearson Chi-Square test. ^2^ In modified pure-tone audiometry at 500 Hz, 97.2% and 98.1% of the subjects had no hearing loss in the right and left ear, respectively. Accordingly, 2.8% and 1.9% had a hearing threshold between 26 and 40 dB in the right and left ear, respectively. These differences between the right and left ear were not significant (*p* > 0.2).

## Data Availability

Data are available from the corresponding author upon reasonable request.
